# STDP-based adaptive graph convolutional networks for automatic sleep staging

**DOI:** 10.3389/fnins.2023.1158246

**Published:** 2023-04-20

**Authors:** Yuan Zhao, Xianghong Lin, Zequn Zhang, Xiangwen Wang, Xianrun He, Liu Yang

**Affiliations:** College of Computer Science and Engineering, Northwest Normal University, Lanzhou, China

**Keywords:** sleep stage classification, graph convolutional network (GCN), spike-timing-dependent plasticity (STDP), domain adaptation, Polysomnography (PSG)

## Abstract

Automatic sleep staging is important for improving diagnosis and treatment, and machine learning with neuroscience explainability of sleep staging is shown to be a suitable method to solve this problem. In this paper, an explainable model for automatic sleep staging is proposed. Inspired by the Spike-Timing-Dependent Plasticity (STDP), an adaptive Graph Convolutional Network (GCN) is established to extract features from the Polysomnography (PSG) signal, named STDP-GCN. In detail, the channel of the PSG signal can be regarded as a neuron, the synapse strength between neurons can be constructed by the STDP mechanism, and the connection between different channels of the PSG signal constitutes a graph structure. After utilizing GCN to extract spatial features, temporal convolution is used to extract transition rules between sleep stages, and a fully connected neural network is used for classification. To enhance the strength of the model and minimize the effect of individual physiological signal discrepancies on classification accuracy, STDP-GCN utilizes domain adversarial training. Experiments demonstrate that the performance of STDP-GCN is comparable to the current state-of-the-art models.

## 1. Introduction

A proper sleep cycle plays a vital role in maintaining one's mental and physical wellbeing. However, with the increasing mental stress of modern life, sleep disorders have become an issue that cannot be overlooked. Sleep quality and sleep disturbances are usually assessed by dividing the sleep state according to the patient's Polysomnography (PSG) throughout the night, PSG records various human physiological signals such as Electroencephalography (EEG), Electromyogram (EMG), Electrooculogram (EOG) and Electrocardiogram (ECG). The Rechtschaffen and Kales standard (Wolpert, 1969) and American Academy of Sleep Medicine (AASM) standard (Berry et al., 2012) are commonly used to classify PSG signals as a standard code for classifying sleep states. One person's overnight PSG recording is a very large amount of data, manually labeling such a large number of PSG signals is a very single and tedious task, and it is prone to errors, which is unbearable for clinically diagnosed sleep disorders. Therefore, it is crucial to identify and categorize sleep state staging in order to properly diagnose sleep-related disorders. Automatic sleep staging can greatly improve the efficiency and accuracy of sleep state classification, and greatly liberate human resources so that experts can focus more on diagnosing and treating diseases.

There has been a lot of valuable work on automatic sleep state classification in recent years, automatic sleep staging mainly uses traditional machine learning methods in the early stage, such as Support Vector Machine (SVM) (Alickovic and Subasi, 2018) or Random Forest (RF) (Memar and Faradji, 2018), which have high requirements for handcrafted features. Since traditional machine learning methods require complex feature engineering, researchers began to use deep learning for automatic sleep staging and achieved high accuracy (Supratak et al., 2017; Phan et al., 2019; Bakker et al., 2022; Li et al., 2022; Martín-Montero et al., 2023; Zhang et al., 2023). Although deep learning methods have achieved high accuracy, they have not fully exploited the topology of functional connections in different brain regions. The brain is a complex network of structurally and functionally interconnected regions, localized dysfunction often propagates and affects other regions leading to large-scale network changes. The recent development of graph neural networks (GNN) (Kipf and Welling, 2016) has led researchers to explore the use of GNN to extract spatial features of PSG signals, the GraphSleepNet (Jia et al., 2020) uses GCN to extract EEG signals by using EEG signals as nodes of GNN, which achieves state-of-the-art performance compared to previous methods.

In addition, the automatic sleep staging task also face a challenge, which is the trained model often performs well on the training data set, but the performance of the model is often unsatisfactory due to individual differences or measurement equipment errors in actual application. The physiological signals of different subjects vary greatly, so it is necessary to consider improving the adaptability of the model to different data distributions. Some efforts have tried to use domain adaptation to improve the adaptability of the model (Tzeng et al., 2014; Ganin et al., 2016; Jia et al., 2021a) and have achieved good results. The basic idea of Domain Adaptation is to map the source domain and target domain data into a feature space. By finding a unified metric in the same feature space, the feature distribution of the source domain and target domain data is as close as possible, which can improve the performance of the model based on source domain data feature training on target domain data.

The current method has achieved high accuracy in automatic sleep staging tasks, but the following challenges still need to be solved: (1) The feature extraction ability of the model needs to be improved. In particular, the current model does not make full use of the functional connection between brain regions and the interdependence between different modes of data in PSG data. (2) The graph-building algorithm of the GNN model is often based on back-propagation while ignoring the interpretability of the graph-building algorithm. (3) It is necessary to effectively improve the adaptive ability of the model to the data. Due to the huge differences in physiological signals between individuals, models with good performance in training data sets often perform poorly in actual deployment.

The establishment method of graph structure is the core to solving the first two challenges, which due to a graph of different brain regions can be seen as an explainable result, as brain region connections with abnormal patterns can help explain the causes of sleep disorders (Griffa et al., 2013). Building an explainable graph structure is difficult due to: (1) Pre-defined graphs cannot adapt to functional connectivity of brain regions in different sleep stages; (2) Graph generation algorithms trained by end-to-end may learn unsuitable parameters with small amounts of train data, and this approach is less explainable.

To address the difficulty of building graphs for GNN, we adaptively compute graph structures through a neuroscience mechanism. When using GNN for automatic sleep staging, we assume that each PSG channel corresponds to a node in the graph, and the connections between channels correspond to connections between different brain regions. The connections between brain regions are made up of connections between neurons, and neurons are connected through synapses, so it is reasonable to build connections between brains through the strength of synapses. The synapses adjustment rule between neurons has made a lot of progress in neuroscience (Fornito et al., 2015), such as the Hebbian theory proposed by Hebbian (Hebb, 1949), which shows that the weight between two neurons increases if the two neurons activate simultaneously, and reduces if they activate separately, which is often summarized as “Cells that fire together wire together". But Hebbian theory doesn't make predictions about the firing of presynaptic neurons after postsynaptic neurons, which is solved by spike-timing-dependent plasticity (STDP). The concept of STDP was first proposed by Taylor (1973), Bi and Poo (1998) discovered that postsynaptic synapses that were activated within 5–20 ms before the spike were strengthened, whereas synapses that were activated within a similar time window after the spike was weakened, STDP core idea is to calculate the weight of the direct connection of two neurons according to the sequence of the two connected neurons firing pulses (Dan and Poo, 1992). These rules about weight adjustment between neurons motivate us to apply weight adjustment rules between neurons to build graph structures.

In this paper, we propose an adaptive GCN based on Spike-Timing-Dependent Plasticity, named STDP-GCN. The connection between various PSG signal channels forms a graph structure, and the channel of the PSG signal may be thought of as a neuron, the STDP process used to build the strength of the synapses between neurons, which builds the graph. The transition rules between sleep stages are extracted using temporal convolution after the GCN, and classification is performed using a fully connected neural network. In particular, domain adaptation is applied in the classification network to improve the adaptive ability of the STDP-GCN. We summarize the main contributions of this paper:

- An explainable STDP adaptive graph learning algorithm is proposed. The STDP adaptive graph learning algorithm employs the STDP mechanism from neuroscience to dynamically establish inter-channel dependencies without any labeling and exhibits exceptional performance.
- The proposed STDP-GCN can capture both temporal and spatial features of PSG separately through spatio-temporal graph convolution. Furthermore, it can reduce discrepancies between individual physiological signals and enhance performance through domain adaption.
- Through comparative experiments on the ISRUC-S3 dataset and SLEEP-EDF-153 dataset, the proposed STDP-GCN demonstrated the highest accuracy compared to existing models.

## 2. Related works

### 2.1. Sleep stage classification problem

The human sleep process can be divided into three main parts: Wake, Rapid Eye Movement (REM), and Non-rapid Eye Movement (NREM) according to AASM standard (Berry et al., 2012). The main features of REM are rapid eye movements and relaxation of body muscles, while NREM is characterized by shallower, slower, and more uniform breathing, slower heart rate, lower blood pressure, and no obvious eyeballs. NREM can be divided into three stages: N1, N2, and N3 to assess the depth of sleep. This article divides sleep states into five categories (Wake, N1, N2, N3, and REM) according to the AASM standard.

The PSG signal is divided into epochs of the 30s, and each epoch is labeled as a sleep state. According to the AASM standard, experts use the features of the PSG data of the current epoch and the previous and previous epochs to mark the sleep state of the current epoch, because sleep state transition patterns are very valuable, for example, it usually enters the N1 stage after wake stage.

In this paper, the sleep stage classification problem can be defined as input multiple epochs, which is defined as $X=\left(x_{i-c},...,x_{i},...,x_{i+c}\right)\in R^{M\times N\times L}$, output a sleep state of the current epoch $\hat{y}$, where *c* indicates the temporal context, and *M* = 2*c* + 1 is the number of temporal contexts, *N* is the number of nodes in the PSG, *L* is the number of features per channel.

### 2.2. Automatic sleep staging methods

Recent years have seen a significant amount of research in the academic field surrounding automatic sleep staging, due to its crucial role in the diagnosis of sleep disorders. Designing features for PSG signals manually through traditional methods is a challenging task due to the complexity of the signal features, which makes deep learning particularly effective in the task of automatic sleep staging.

With the rapid development of deep learning, Convolutional Neural Networks (CNNs) and Recurrent Neural Networks (RNNs) are widely used in automatic sleep staging. Zhang and Wu (2017) propose a new model called Fast Discriminant Complex-valued Convolutional Neural Network (FDCCNN) for extracting features from raw EEG data and classifying sleep stages. Chambon et al. (2018) introduced a deep neural network to perform temporal sleep stage classification from multimodal and multivariate time series, which can be learned end-to-end without computing spectrograms or extracting manual features. Phan et al. (2019) propose a hierarchical recurrent neural network named SeqSleepNet, which is designed to run on multi-channel time-frequency image inputs to solve the automatic sleep staging problem. Perslev et al. (2019) propose U-time to analyze physiological time series segmentation of sleep data. Cai et al. (2021) propose a novel graph-time fusion dual-input convolutional neural network approach to detect sleep stage. Perslev et al. (2021) introduce A deep learning-based automated sleep staging system (U-SLEEP) that provides accurate segmentation of A wide range of patient cohorts and PSG protocols that were not considered when building the system. Jia et al. (2021b) propose the SalientSleepNet, which is a multimodal significant wave detection network for sleep staging.

Although deep learning achieves high performance, it ignores the interdependencies between PSG signal channels. Jia et al. (2020) propose a new deep graph neural network GraphSleepNet for automatic sleep stage classification, which can adaptively learn the internal connections between different EEG channels. Thus, it can better serve the spatio-temporal graph convolution network (ST-GCN) for sleep stage classification. The lack of interpretability in the above methods highlights the need for a model with explainable features, as interpretability is crucial for understanding the underlying cause of sleep disorders in neuroscience.

## 3. Methodology

**Figure 1:**
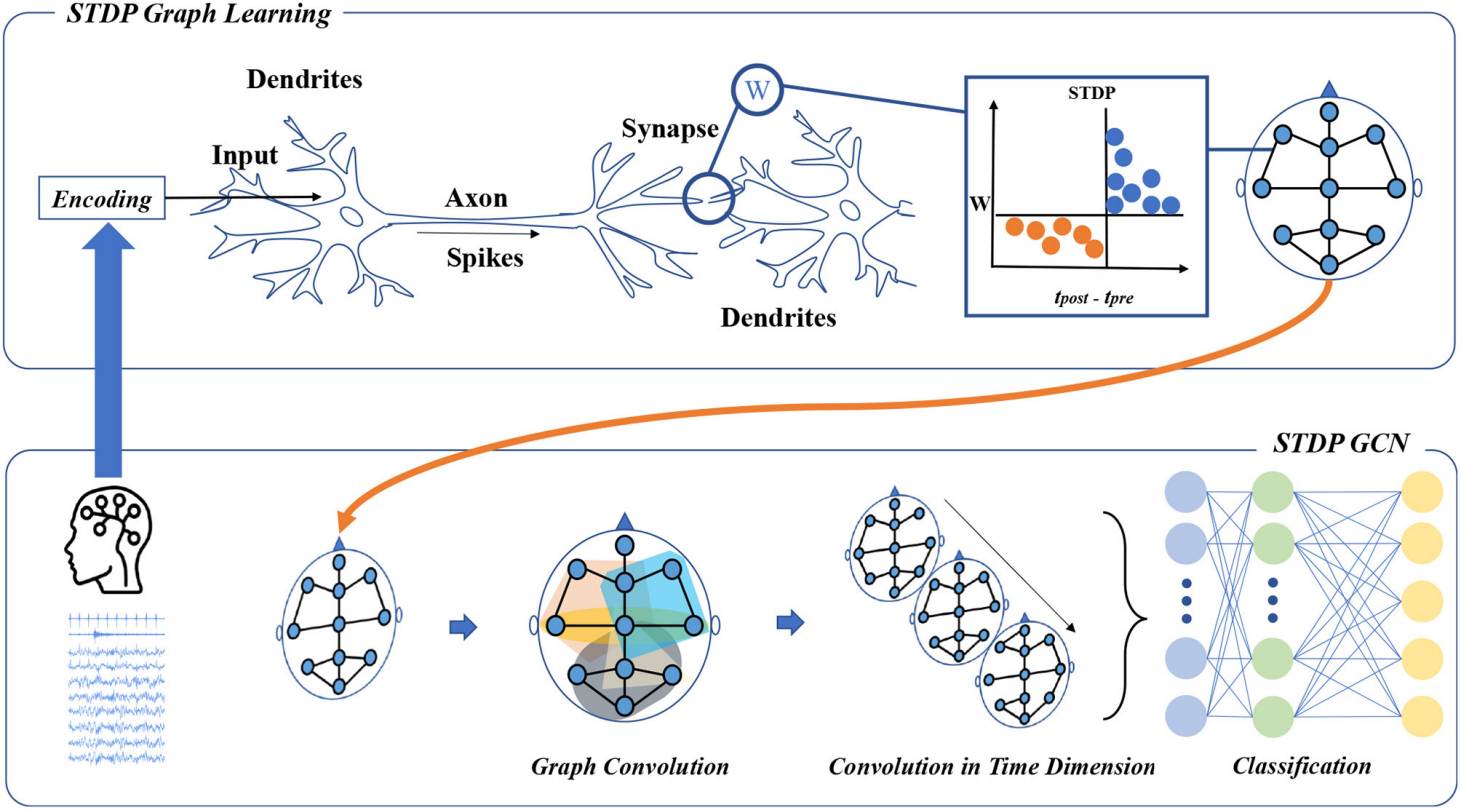
The framework of STDP-GCN. The STDP graph learning approach views each channel in the PSG as a neuron, with the STDP mechanism determining the strength of connections between neurons to form a graph structure across the PSG channels. The graph structure is then used for graph convolution, followed by temporal convolution to learn the sleep stage transition rules, and a fully connected neural network is applied for classification.

The overall architecture of STDP-GCN is shown in Figure 1. The main ideas of STDP-GCN are as follows: (1) Build the graph structure using an adaptive STDP graph learning algorithm; (2) After a spatio-temporal GCN aggregates the signal, and a fully connected network is used for classification. Models are carefully designed to get the best results in this paper.

### 3.1. STDP Graph Learning

The process of STDP graph learning algorithm is: (1) encode PSG signals into pulse sequences; (2) calculate the connection weights between pulse sequences according to STDP algorithm, so as to obtain the interdependence between PSG channels. This section first introduces the encoding algorithm and STDP algorithm, and then introduces the STDP graph learning algorithm.

**Figure 2:**
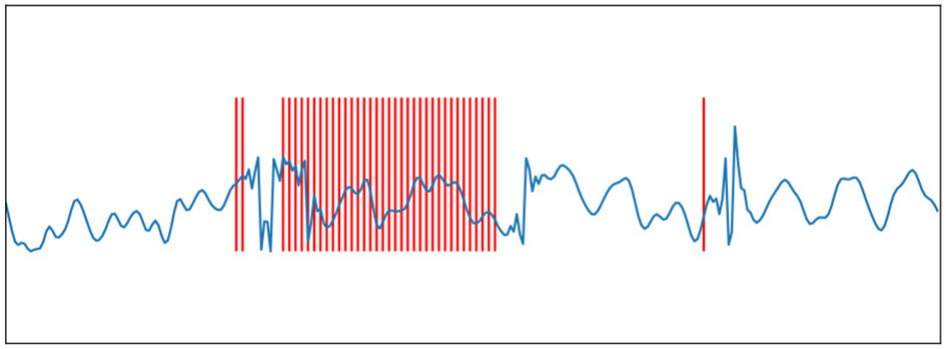
Ben's Spiking Algorithm. The blue curve is an EEG signal, and the red vertical line is the spike sequence encoded by the EEG signal according to the BSA encoding algorithm.

**Figure 3:**
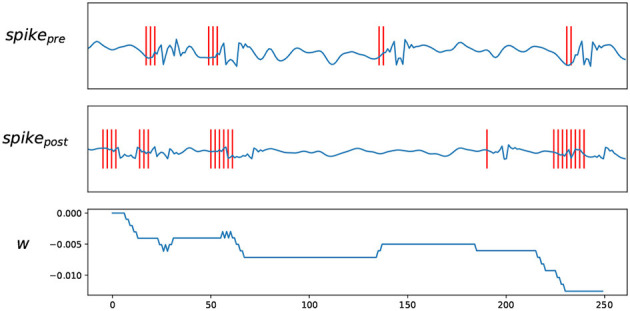
Trace STDP rules. *spike*_*pre*_ and *spike*_*post*_ are the EEG signals and their spike trains encoded by BSA, *W* shows how the weights change according to the spike trains.

**Encoding:** STDP learning needs the spike train as input, so the raw PSG signal needs to be converted into a spike train at first. Encoding continuous signals is typically accomplished using analog-to-spike encoding algorithms, including Threshold Based Representation (TBR), Ben's Spiking Algorithm (BSA) (Schrauwen and Van Campenhout, 2003), and Moving Window (MW) (Petro et al., 2020). Typically, BSA algorithms are employed to transform audio data into a spike train. However, as PSG signals are also distributed across the frequency spectrum, some studies (Nuntalid et al., 2011; Medini et al., 2015). have utilized BSA algorithms to encode PSG signals. So the BSA algorithm is well suited to encode PSG signals. In this paper, the BSA algorithm is used to convert the PSG signal into a spike train. The BSA algorithm is based on encoding a signal using an FIR filter. The Finite Impulse Response (FIR) filter is widely used in digital signal processing, the main function is to leave a useful signal, we set the cutoff frequency of FIR to 0.8 and the length to 20 according to the BSA algorithm. By computing two error values Eq.(1) and Eq.(2) at each time instant *τ*, which can be defined as


(1) $$\begin{array}{l} error1=\sum_{k=0}^{M}abs\left(s\left(k+\tau \right)-h\left(k\right)\right), \end{array}$$

(2) $$\begin{array}{l} error2=\sum_{k=0}^{M}abs\left(s\left(k+\tau \right)\right), \end{array}$$

here *s* is the original signal and *h* is an FIR filter of length *M*. If Eq.(1) is less than Eq.(2) minus the threshold, then encode a spiking and subtract the filter from the input. The signal can be recovered from the spike train by a convolution between the spike train and the FIR filter. The origin signal and its spike train encoded using BSA are shown in Figures 2, 3.

**Spike-timing-dependent plasticity:** After encoding the PSG signal into a spike train, the STDP algorithm is used to learn the correlation between the pulse sequences. STDP learning rules are a synaptic plasticity mechanism discovered in biological experiments (Bi and Poo, 1998). A typical neuron consists of a cell body (soma), dendrites, and a single axon. Dendrites receive action potentials from other neurons and transmit them to the body of the cell. Axon's function is to transmit information to different neurons.

Under the STDP process, the synapse will strengthen if the firing spike of the pre-neuron tends to occur on average before the output spike of the post-neuron. If the spiking of the pre-neuron tends to occur immediately after the output spiking of the post-neuron, the synapse weights of the two neurons are slightly weaker (Bi and Poo, 1998). In general, the STDP process can be defined as


(3) $$\text{Δ}\omega =\left\{ \begin{array}{l} \;Ae^{\frac{t_{pre}-t_{post}}{\tau }},t_{pre}-t_{post}<0,\\ Be^{-\frac{t_{pre}-t_{post}}{\tau }},t_{pre}-t_{post}>0, \end{array}\right.$$

here, Δ*ω* represents the amount of change in synaptic strength, *t*_*pre*_−*t*_*post*_ represents the time difference between the presynaptic pulse and the postsynaptic pulse, *A* > 0 and *B* < 0 are the learning rates that control the Δ*ω*. However, the implementation of Eq. (3) is not feasible as it requires separate recording of the firing times of neurons before and after. It is easier to implement STDP using the double-pulse trace-based approach (Morrison et al., 2008) provided by Eq.(4), Eq.(5). The core idea of the double-pulse trace-based approach is that synaptic weights decrease when the pre-neuron fired spike and the synaptic weight increases when the post-neuron is fired.


(4) $$\begin{array}{l} \text{Δ}\omega_{ij}^{-}\left(t_{j}^{f}\right)=-F_{-}\left(\omega_{ij}\right)y_{i}\left(t_{j}^{f}\right), \end{array}$$

(5) $$\begin{array}{l} \text{Δ}\omega_{ij}^{+}\left(t_{i}^{f}\right)=-F_{+}\left(\omega_{ij}\right)y_{i}\left(t_{i}^{f}\right), \end{array}$$

here, Eq.(4) depicts a decrease in synaptic weight when a spike $t_{j}^{f}$ from pre-neuron *j* arrives; Eq.(5) expresses an increase in synaptic weight when a spike $t_{i}^{f}$ from post-neuron *i* arrives, −*F*_+_(*ω*_*ij*_) and −*F*_−_(*ω*_*ij*_) are functions that control the increment of weights.


(6) $$\begin{array}{l} \frac{dx_{j}}{dt}=-\frac{x_{j}}{\tau_{x}}+\underset{t_{j}^{f}}{\sum }\delta \left(t-t_{j}^{f}\right), \end{array}$$

(7) $$\begin{array}{l} \frac{dy_{i}}{dt}=-\frac{y_{i}}{\tau_{y}}+\underset{t_{i}^{f}}{\sum }\delta \left(t-t_{i}^{f}\right). \end{array}$$

The double-pulse trace-based approach uses trace to describe pre-neuron membrane potential *x*_*j*_ and post-neuron membrane potential *y*_*i*_. The membrane potential rises immediately when the neuron receives a spike, and then slowly decreases to the resting potential over time, which can be expressed by differential equation Eq.(4), Eq.(5). $t_{j}^{f}$ and $t_{i}^{f}$ is the spike firing time of post-neuron *i* after pre-neuron *j*, *δ* is the pulse function, which is 1 at *t* = 0, and 0 at other times.

**Adaptive graph learning:** In this paper, the PSG signal input of an epoch is defined as a graph *G*(*V, E, A*), where *V* is the set of nodes in the graph, each node corresponds to a channel in the PSG, and *E* represents the edge between nodes, *A* is the adjacency matrix of the graph. The adjacency graph is an important input of the graph neural network. In this paper, the graph learning algorithm can be defined as inputting PSG data of an epoch and outputting a graph structure of the epoch.

The main purpose of adaptive STDP graph learning is to learn graph structures using STDP. As shown in the upper part of Figure 1, when the PSG is input to the STDP adaptive graph learning module, the signals of each channel in the PSG are first encoded into a spike train by the BSA algorithm. The spike train of a channel is regarded as the spike train emitted by a neuron, the connection between the channel and the channel can be regarded as a synapse, and the synapse strength can be obtained by the STDP. If the pre-neuron emits a spike before the post-neuron emits a spike, it can be seen that there is a connection between the pre-neuron and the post-neuron. The STDP graph learning algorithm used in this paper is distinct from other graph structure construction algorithms in that it relies on the STDP algorithm to establish interdependencies between different channels. However, this algorithm requires time steps for simulation, resulting in increased computational overhead. To save time, we employ an improved STDP algorithm and GPU parallel computing. After the STDP graph learning module, the relationship between channels and channels can be obtained, which is represented as an adjacency matrix. The topology of multivariate data can be used as input to spatio-temporal graph convolution to extract feature representations in the spatial dimension.

The input of STDP-GCN is a sequence of multiple epochs, each epoch will use the STDP graph algorithm to adaptively learn a graph structure, which can be defined as input *X* = (*x*_*t*−*c*_, ..., *x*_*t*_, ..., *x*_*t*+*c*_), output *A* = (*a*_*t*−*c*_, ..., *a*_*t*_, ..., *a*_*t*+*c*_). The PSG signal and graph structure at time step *t* are represented by *x*_*t*_ and *a*_*t*_, respectively. The weight calculation between channel *j* and channel *i* in the STDP graph structure algorithm can be expressed as


(8) $$a_{ji}=\sum_{t=1}^{L}i(\text{Δ}\omega_{ij}^{-}\left(t_{j}^{f}\right)+\text{Δ}\omega_{ij}^{+}\left(t_{j}^{f}\right)),$$

here, *a*__*ji*__ is the synapse weight between pre-neuron and post-neuron, $\text{Δ}\omega_{ij}^{-}\left(t_{j}^{f}\right)$ is the amount of change in the synaptic weight when the presynaptic spike is fired, and $\text{Δ}\omega_{ij}^{+}\left(t_{i}^{f}\right)$ is the amount of change in the synaptic weight when the post-synaptic fired spike. After constructing the graph, the preprocessed original signal and the adjacency graph enter the STGCN layer together.

The cross-entropy is used as a loss function to tune the parameters of the spatio-temporal graph convolution, which is defined as


(9) $$\begin{array}{l} L=-\frac{1}{L}\overset{L}{\underset{i=1}{\sum }}\overset{N}{\underset{n=1}{\sum }}y_{i,n}\log\left(\hat{y}\right), \end{array}$$

here, *L* is the number of samples, while *N* is the number of categories of sleep stages, and *y* is the ground truth label.

### 3.2. Spatial-temporal graph convolution

**Graph convolution:** The main purpose of graph convolution is to aggregate and extract the spatial dimension features of signals. EEG of different channels can measure the electrical signals of corresponding brain regions, and the relationship between signals between brain regions can be aggregated by graph convolution. We use spectral graph convolution theory to build graph convolution layers and to speed up training, we use a simplified GCN. The signal propagation between layers is shown in Eq.(10), where $D^{-\frac{1}{2}}AD^{-\frac{1}{2}}$ is the constructed Laplacian matrix, *A* is the adjacency graph matrix constructed by STDP, *H* is the result of the previous layer, and *W* is the learnable parameter matrix, σ is the activation function.


(10) $$\begin{array}{l} H^{\left(l\right)}=\sigma \left(D^{-\frac{1}{2}}\;A\;D^{-\frac{1}{2}}H^{\left(l-1\right)}W^{\left(l-1\right)}\right). \end{array}$$

**Convolution in time dimension**: According to the AASM standard, the sleep transition rule, that is, the sleep staging of the preceding and following periods is an important reference condition for judging the current sleep state. Therefore, taking transition rules into account can improve the accuracy of the classification. STDP-GCN utilizes an adaptive STDP graph learning algorithm for graph construction and feature extraction through GCN at distinct time steps. It subsequently employs time-wise convolution to learn the transition rules. After the data passes through the graph convolution layer, the information of the data has been fully aggregated, and then convolution in the time dimension will better extract the sleep transition rules. The convolution in the time dimension in this paper can be described as follows:


(11) $$\begin{array}{l} H^{\left(l+1\right)}=softmax\left(\text{Φ}*\left(softmax\left(H^{\left(l\right)}\right)\right)\right), \end{array}$$

here *softmax* is the activation function, Φ denotes the convolution kernel, * denotes the standard convolution operation.

**Domain adaptation**:Machine learning models rely heavily on data distribution and the data distribution of PSG may vary significantly due to individual differences. Therefore, we hope that STDP-GCN can effectively learn how to extract common core features. By treating an individual's physiological signal as a domain, we can use the domain adaptation to learn the common features between domains and effectively improve the robustness of the model.

The idea of domain adversarial training (Ganin et al., 2016) originates from Generative Adversarial Network (GAN) (Goodfellow et al., 2014), which consists of a generator and a discriminator. Generators are used to generate false data, and discriminators are used to determine whether the input data is generated false data or real data. The core idea of GAN is to hope that the false data generated by the generator can deceive the discriminator, which is also improving the discriminant ability to prevent being deceived. The two play against each other until the whole system reaches a stable state. Similarly, domain adversarial training is when the model extracts features from the source domain and the target domain, respectively, and then trains the discriminator, hoping that the discriminator cannot distinguish the extracted features from the source domain from the target domain. This allows the target domain's data to be generated with a feature distribution as close to the source image as possible, thereby reducing the domain shift.

**Figure 4:**
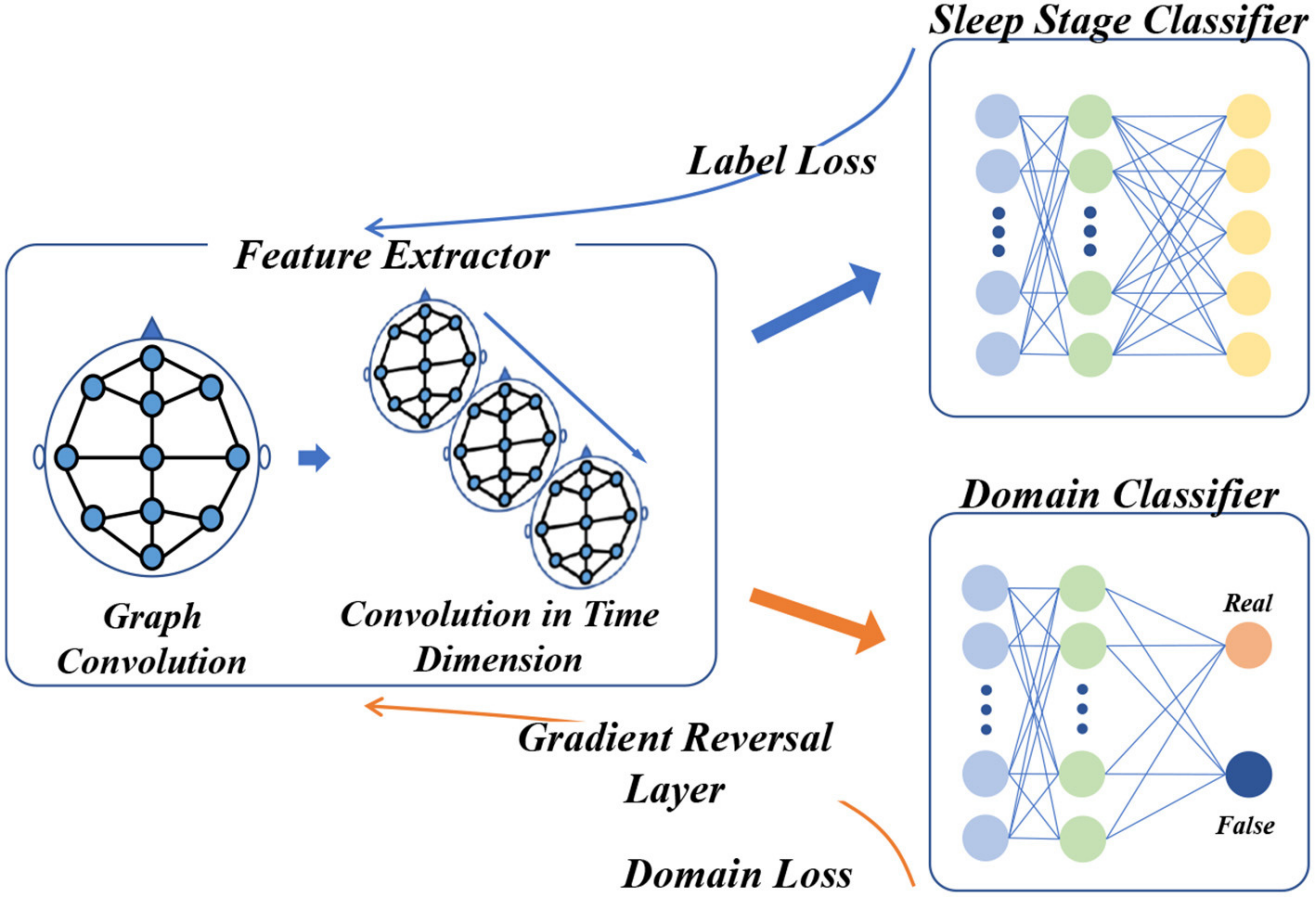
Domain adversarial training framework for STDP-GCN. Following the blue arrow, the PSG signal is processed through the feature extractor to acquire the domain-invariant feature, which then proceeds to the sleep stage classifier for classification and computation of the label loss, before undergoing back-propagation. Along the yellow arrow, the domain-invariant feature is fed into the domain discriminator for domain classification and calculation of the domain loss, which then undergoes backpropagation through the Gradient Reverse Layer.

As depicted in Figure 4, there are two main tasks to be completed in the domain adversarial training of STDP-GCN: (1) Accurate classification of source domain datasets to minimize the error of automatic sleep staging; (2) To confuse the source domain dataset with the target domain dataset, maximize the domain classification error. Feature extractor *G*_*f*_ maps input *x*_*i*_ to feature space to get domain-invariant feature *X*_*f*_, and then *X*_*f*_ input domain discriminator *D* and sleep stage classifier. Feature extractor *G*_*f*_ is defined as


(12) $$\begin{array}{l} X_{f}^{i}=G_{f}\left(x_{i};\theta_{f}\right), \end{array}$$

where $X_{f}^{i}$ denotes the transferred features of *x*_*i*_, *θ*_*f*_ is the trainable parameter of *G*_*f*_.

Sleep stage classifier *G*_*f*_ and its loss can be defined as


(13) $$\begin{array}{l} {\hat{y}}_{c}^{i}=G_{y}\left(X_{f}^{i};\theta_{y}\right) \end{array}$$

(14) $$\begin{array}{l} L_{c}\left({\hat{y}}_{c}^{i},y_{i}\right)=\log\frac{1}{{\hat{y}}_{c}^{i}}y_{i} \end{array}$$

where ${\hat{y}}_{i}^{c}$ predicted label, *θ*_*y*_ is the trainable parameter of *G*_*y*_. Domain discriminator *G*_*d*_ and its loss can be defined as


(15) $$\begin{array}{l} {\hat{y}}_{d}^{i}=G_{d}\left(X_{f}^{i};\theta_{d}\right) \end{array}$$

(16) $$L_{d}({\hat{y}}_{d}^{i},d_{i})=d_{i}\log\frac{1}{{\hat{y}}_{d}^{i}}+(1-d_{i})\log\frac{1}{{\hat{y}}_{d}^{i}}$$

where ${\hat{y}}_{i}^{d}$ is the predicted result of the domain discriminator, *d*_*i*_ represents the binary label of the *i*-th sample and is used to indicate whether the sample belongs to the source or target domain, *θ*_*y*_ is the trainable parameter of *G*_*y*_. The overall loss of training can be defined as


(17) $$\begin{array}{l} E\left(\theta_{f},\theta_{c},\theta_{d}\right)=\frac{1}{n}\overset{n}{\underset{i=1}{\sum }}L_{c}^{i}-\text{λ}\left(\frac{1}{n}\overset{n}{\underset{i=1}{\sum }}L_{d}^{i}+\frac{1}{n_{i}^{\prime }}\overset{n}{\underset{i=n+1}{\sum }}L_{d}^{i}\right) \end{array}$$

Through Gradient Reversal Layer (GRL), domain adaptation can be naturally integrated into the back-propagation algorithm of the network to unify the training process. The network optimization process is defined as


(18) $$\begin{array}{l} \left({\hat{\theta }}_{f},{\hat{\theta }}_{c}\right)=\text{arg}\underset{{\hat{\theta }}_{f},{\hat{\theta }}_{c}}{\min}L\left(\theta_{f},\theta_{c},{\hat{\theta }}_{d}\right) \end{array}$$

(19) $$\begin{array}{l} {\hat{\theta }}_{d}=\text{arg}\underset{\theta_{d}}{\min}L\left({\hat{\theta }}_{f},{\hat{\theta }}_{y},\theta_{d}\right) \end{array}$$

The parameters of the sleep stage classifier are updated by minimizing the objective function, and the parameters of the domain discriminator are updated by maximizing the objective function.

## 4. Experiments

### 4.1. Datasets and experimental settings

In this paper, the ISRUC-S3 dataset and SLEEP-EDF-153 dataset are used to verify the validity of the STDP-GCN model. There are PSG recordings of 10 healthy subjects in the ISRUC-S3 dataset, with 6 EEG channels, 2 EOG channels, 3 EMG channels, and 1 ECG channel. Each epoch is divided into 5 sleep states by AASM standard. The SLEEP-EDF-153 dataset recorded the PSG signals of 78 healthy subjects, and the EEG was obtained by sampling from the Fpz-Cz and Pz-Oz electrode positions at 100 HZ. The SLEEP-EDF-153 dataset classifies labels into eight modes (wake-up, S1, S2, S3, S4, REM, motion, and unknown) according to the Rechtschaffen and Kales standard (Wolpert, 1969). To simplify the process of setting experimental parameters, we combine S3 and S4 into S3 according to AASM standards (Berry et al., 2012).

We use subject-independent cross-validation to test the effect of the STDP-GCN. Due to the different number of individuals contained in the data set, we apply 10-fold cross-validation on the ISRUC-S3 data set and 20-fold cross-validation on the SLEEP-EDF-153 dataset. The hyperparameters of STDP-GCN are listed in Table 1, and we apply the same experimental settings to all baselines to pursue comparative fairness. This article uses PyTorch to implement the model and training, and the code has been released at: https://github.com/thegoist/STDP-GCN.

**Table 1:** Experiment hyperparameter setting.

**Hyperparameter description**	**Value**
Optimizer	Adam
Learning rate	1e-4
Number of training epochs	500
Batch size	256
Dropout probability	0.5
Weight decay	1e-3
Layer number of GCN	1
The number of temporal contexts *M*	5
*τ* values of neurons	100.0
Threshold for spiking	1.0
Learning rate for STDP	1e-2
Domain classifier architecture	450-512-100-2
Initial λ value of Gradient Reversal Layer	1.0

### 4.2. Experimental results and comparison

This section uses STDP-GCN to compare with the other baselines, showing the superiority of the current STDP-GCN. As evident from Tables 2, 3, STDP-GCN outperforms prior methods in multiple metrics. By utilizing the STDP mechanism in constructing its graph structure, STDP-GCN aligns with the principles of neuroscience and effectively leverages the inter-channel dependencies to enhance the extraction of spatial features, resulting in better performance across various indicators. It can be observed from the table that the traditional machine learning algorithm SVM and RF is less accurate than other methods because it cannot learn temporal transition rules, while CNN and RNN can rely on learning transition rules in the time dimension and learning features in the spatial dimension to achieve higher accuracy. The channels in the PSG signal are not separated by Euclidean distances, so using Euclidean distance for convolution may overlook the non-Euclidean distance information between channels. The experimental data demonstrates that Wake and N1 indicators are always mutually exclusive. An increase in the Wake indicator leads to a decrease in the N1 indicator. The reason behind this is that the N1 stage is prone to misclassification as Wake due to the shared characteristics between them. Based on the AASM standard (Berry et al., 2012), both fully awake and drowsiness are included in the Wake stage, and the electrophysiological signals and psychological characteristics of drowsiness even continue to the N1 stage, which could be the main reason for misclassification. In addition, we also explored the effect of different folds on cross-validation, as shown in Table 4. We also applied 5-fold cross-validation on ISRUC-S3, where the performance of 5-fold cross-validation decreased relative to 10-fold, probably due to the increase in the adversarial sample and the decrease in the test sample.

**Table 2:** Overall results comparison on ISRUC-S3.

**Methods**	**Overall results**		**F1-score for each class**				
	**Accuracy**	**F1-score**	**Wake**	**N1**	**N2**	**N3**	**REM**
SVM (Alickovic and Subasi, 2018)	73.3%	72.1%	86.8%	52.3%	69.9%	78.6%	73.1%
RF (Memar and Faradji, 2018)	72.9%	70.8%	85.8%	47.3%	70.4%	80.9%	69.9%
MLP+LSTM (Dong et al., 2018)	77.9%	75.8%	86.0%	46.9%	76.0%	87.5%	82.8%
CNN+BiLSTM (Supratak et al., 2017)	78.8%	77.9%	88.7%	60.2%	74.6%	85.8%	80.2%
CNN (Chambon et al., 2018)	78.1%	76.8%	87.0%	55.0%	76.0%	85.1%	80.9%
ARNN+RNN (Phan et al., 2019)	78.9%	76.3%	83.6%	43.9%	79.3%	87.9%	86.7%
STGCN (Jia et al., 2020)	79.9%	78.7%	87.8%	57.4%	77.6%	86.4%	84.1%
MSTGCN (Jia et al., 2021a)	82.1%	80.8%	**89.4**%	59.6%	80.6%	**89.0**%	85.6%
STDP-GCN	**82.6**%	**81.0** %	83.5%	**62.9**%	**83.1**%	86.0%	**90.6**%

The bold result is the best result.

**Table 3:** Overall results comparison on SLEEP-EDF-153.

**Methods**	**Overall results**		**F1-score for each class**				
	**Accuracy**	**F1-score**	**Wake**	**N1**	**N2**	**N3**	**REM**
SVM (Alickovic and Subasi, 2018)	71.2%	57.8%	80.3%	13.5%	79.5%	57.1%	58.7%
RF (Memar and Faradji, 2018)	72.7%	62.4%	81.6%	23.2%	80.6%	65.8%	60.8%
CNN+BiLSTM (Supratak et al., 2017)	78.5%	75.3%	91.0%	47.0%	81.0%	69.0%	79.0%
MSTGCN (Jia et al., 2021a)	86.4%	**84.1**%	85.5%	**75.3**%	**89.8**%	80.4%	89.3%
STDP-GCN	**87.4**%	83.2 %	**91.1**%	60.1%	89.1%	**84.6**%	**88.8**%

The bold result is the best result.

**Table 4:** Cross-validation of different fold numbers on the ISRUC-S3 dataset.

	**Overall results**		**F1-score for each class**				
	**Accuracy**	**F1-score**	**Wake**	**N1**	**N2**	**N3**	**REM**
5-folds	80.3%	78.5%	83.6%	58.8%	82.0%	82.0%	84.6%
10-folds	82.6%	81.0%	83.5%	62.9%	83.1%	86.0%	90.6%

### 4.3. Experiments and analysis

**Figure 5:**
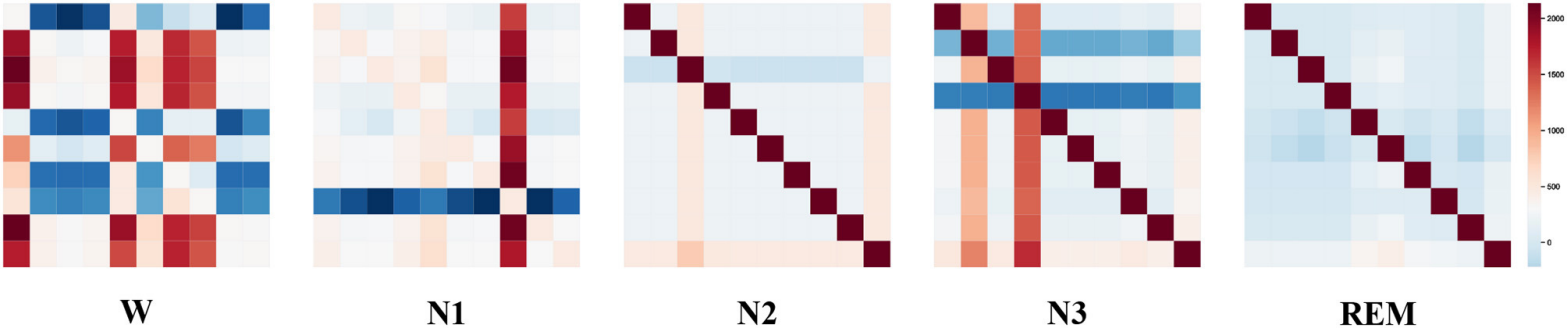
Visualization of the adjacency matrix learned by the STDP graph learning algorithm(Wake Stage, N1 Stage, N2 Stage, N3 Stage, and REM Stage).

To visualize the graph structure generated by the STDP graph learning algorithm, we applied the algorithm to generate the adjacency graph structure of all data in the ISRUC-S3 dataset. By summing up all the adjacency graph matrices adaptive learned through the STDP graph learning algorithm in each state, the brain functional connectivity in each sleep state is shown in Figure 5. The explainability of STDP-GCN can be explored by observing the graph structure generated by the STDP graph learning algorithm. There are numerous functional connections because the brain is more active during the wake period (Larson-Prior et al., 2011). During the NREM stage, the brain gradually enters a deep sleep state and exhibits limited connectivity, typically represented by one or two channels. Conversely, in the REM stage, the functional connections between brain regions are relatively weak (Spoormaker et al., 2010).

**Figure 6:**
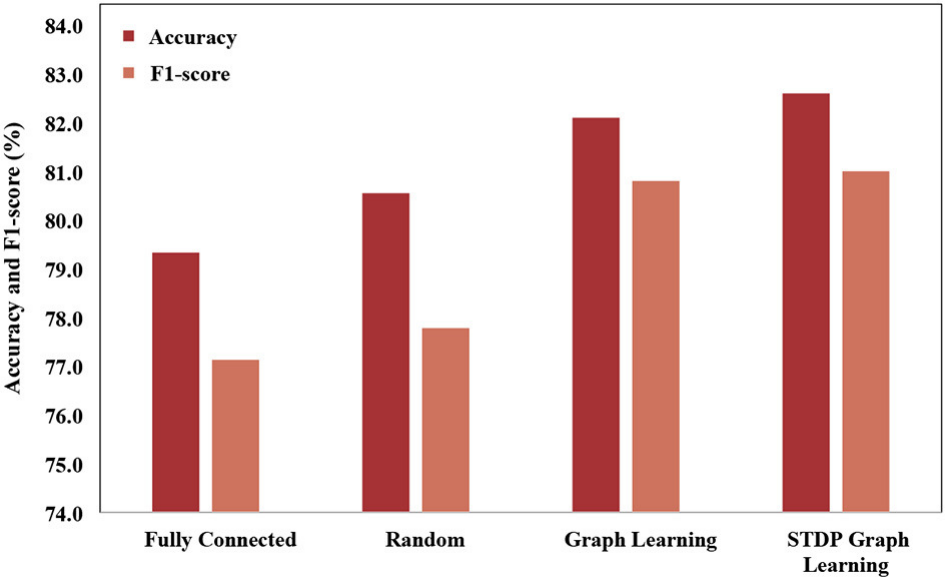
Performance comparison of different graphs.

In order to verify the effectiveness of the STDP graph learning algorithm, this paper uses different graph construction methods to compare the graph structures, which is shown in Figure 6. The graph structures used for comparison mainly include (1) Fully connected adjacency matrix. Fully connected adjacency matrix means that each brain area has functional connections with the same weight, which is not conducive to extracting the spatial features of the graph. (2) Random matrix, each brain area is randomly connected. (3) Graph learning algorithm, which builds the loss by establishing the feature difference between different channels, and learns through backpropagation. Through the experimental results, it can be seen that the fully connected adjacency matrix has the worst effect, and the graph learning algorithm has the best effect, while the STDP graph learning algorithm is close to the graph learning algorithm, and is better than the random matrix algorithm, which it is shown that the graph learned by the STDP graph learning algorithm is effectiveness, and it also shows that the relationship between brain regions can be constructed through synaptic plasticity. The reason why the STDP graph learning algorithm is slightly lower than the graph learning algorithm may be that the STDP algorithm only pays attention to the changes in the synaptic strength caused by the impulse signal between neurons, and the connections between other brain areas are not fully utilized, such as adjacent brain areas. There should also be some connection between the zones.

**Figure 7:**
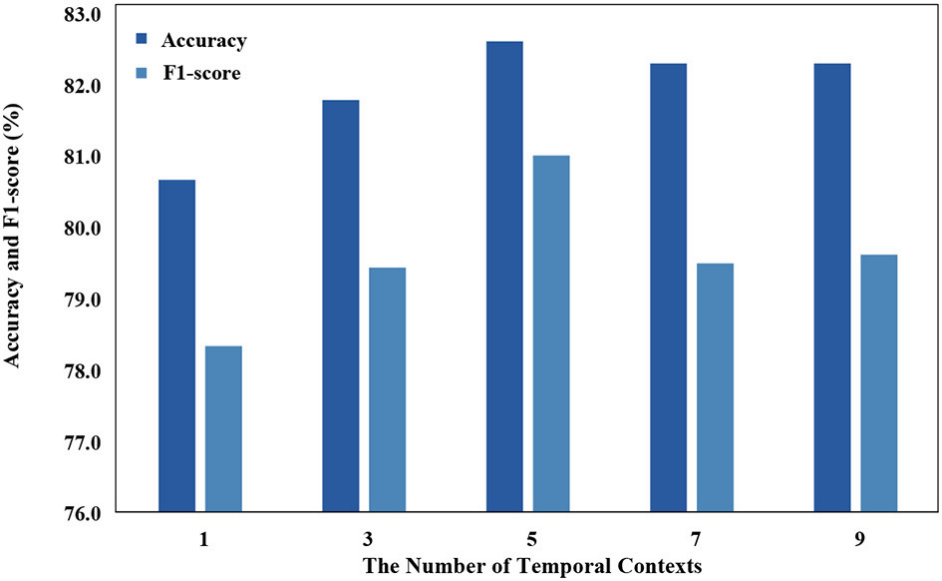
Performance comparison of different time contexts.

Temporal context is used as an input that has a significant impact on the model, and we use different temporal contexts to test their impact on performance. As demonstrated in Figure 7, the classification performance of STDP-GCN on the ISRUC-S3 dataset varies with the number of input contexts *M*. With insufficient input contexts, the model will struggle to learn the temporal transition rules, while an excessive number of contexts will make it challenging for the model to accurately comprehend the temporal transition rules. Optimal performance has been observed when the number of input contexts *M* = 5.

**Figure 8:**
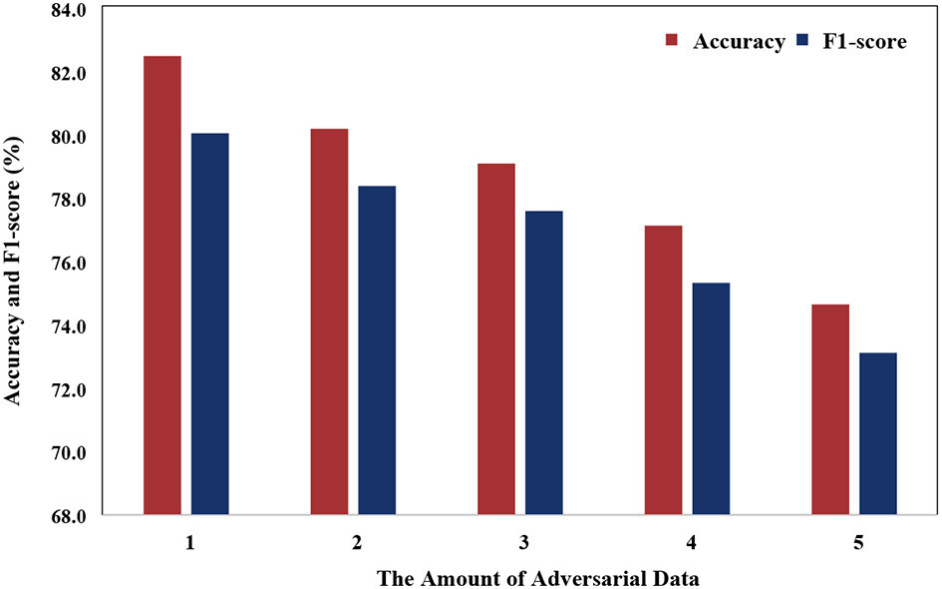
Comparing performance with varying numbers of adversarial data.

Figure 8 illustrates that the model's performance gradually decreases as the number of adversarial data increases. This phenomenon can be attributed to the variance in data distribution of PSG, which is influenced by individual differences. As the number of adversarial samples increases, the number of non-adversarial samples decreases, causing the model to face challenges in learning common features.

**Figure 9:**
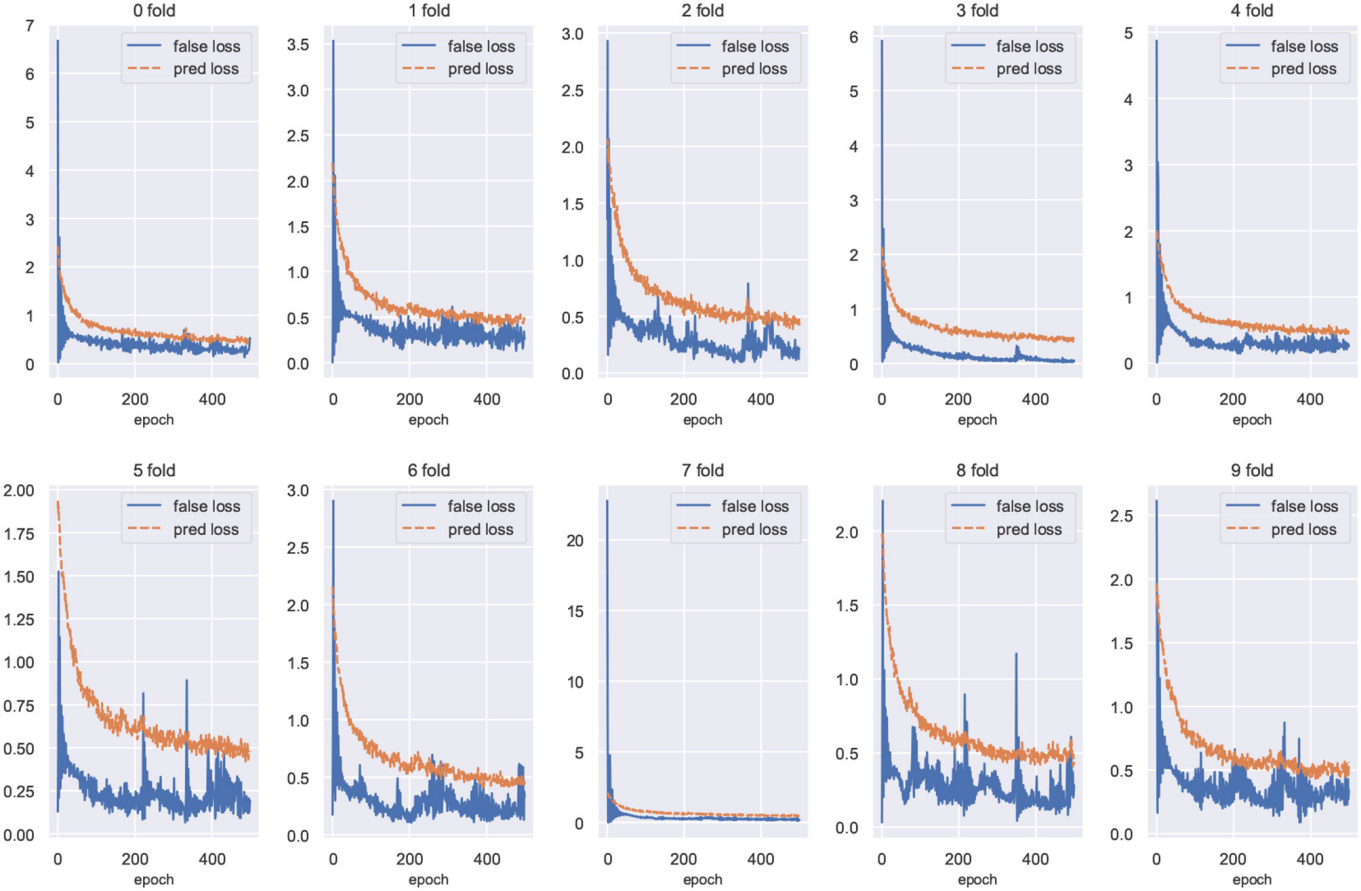
Training loss curves for cross-validation of the ISRUC-S3 dataset. The loss value of the domain discriminator is represented by the blue curve, and the loss of the domain classifier is represented by the red curve.

We plot the training loss curves for subject-independent cross-validation of the ISRUC-S3 dataset. As shown in Figure 9, the loss of the domain classifier decreases and converges as the epoch increases, while the loss of the domain discriminator oscillates but eventually decreases and converges, suggesting that adversarial training is helping the model learn invariant features between domains.

## 5. Discussion

In this paper, we propose STDP-GCN for automatic sleep staging. The main advantage of STDP-GCN is to compute the interdependencies between nodes using the STDP algorithm with neuroscience mechanism, and then construct the graph structure between nodes, so STDP-GCN makes full use of the interdependencies between nodes through GCN to extract features. The STDP graph learning algorithm does not require backpropagation and labeling, it only needs to encode the PSG signal as a pulse sequence to calculate the graph structure of the PSG channel, which not only has a neuroscience mechanism but also has a good performance. As shown in Figure 6, when compared with other graph structure construction algorithms, the STDP graph learning algorithm had the highest accuracy metrics on both the ISRUC-S3 dataset and the SLEEP-EDF-153 dataset, and most of the remaining evaluation metrics outperformed existing methods. In automated sleep staging, individual differences in physiological signals often result in models that perform well in training and poorly in testing. This problem can be effectively addressed by using adversarial training. Figure 9 shows the loss curves of the domain classifier and the domain discriminator during adversarial training on the ISRUC-S3 dataset, from which it can be seen that the loss curve of the domain discriminator decreases in oscillation. This phenomenon indicates that the domain discriminator acts as an adversarial training operation, and in addition, the performance metrics of the model training also prove the effectiveness of adversarial training.

STDP-GCN also comes with some disadvantages. Firstly, the STDP algorithm requires time steps for simulation, and even with the modified STDP algorithm and GPU parallel computing, the STDP graph learning algorithm is still slower than the rest of the graph structure algorithms. Second, STDP-GCN sometimes misclassifies Wake and N1 because both Wake and N1 have similar features. This still indicates that STDP-GCN needs to strengthen its feature learning capability.

## 6. Conclusion

Inspired by Spike-Timing-Dependent Plasticity, this paper proposes an adaptive graph convolution network (GCN) for automatic sleep staging, named STDP-GCN. The key advantage of STDP-GCN is its ability to establish connections between brain regions through the synaptic weight adjustment mechanism among neurons. This algorithm dynamically establishes inter-channel dependencies without any labeling and exhibits exceptional performance. Comparative experiments show that the performance of STDP-GCN is comparable to the leading models in the field.

## Data availability statement

Publicly available datasets were analyzed in this study. This data can be found here: https://sleeptight.isr.uc.pt/; https://physionet.org/content/sleep-edfx/1.0.0.

## Author contributions

YZ wrote the paper and performed the experiment. XL guided the experiment design and reviewed the manuscript. ZZ contributed significantly to the experiment. XW discussed about the results and analysis. XH performed the analysis. LY helped perform the analysis with constructive discussions. All authors helped with developing the concepts and writing the paper. All authors contributed to the article and approved the submitted version.
